# Immunomodulatory effects of heat-killed *Enterococcus faecalis* TH10 on murine macrophage cells

**DOI:** 10.1002/mbo3.41

**Published:** 2012-09-30

**Authors:** Tomohiro Itoh, Yasuyoshi Miyake, Ayumi Onda, Junko Kubo, Masashi Ando, Yasuyuki Tsukamasa, Muneaki Takahata

**Affiliations:** 1 Laboratory of Aquatic Food Science, Department of Fisheries, Faculty of Agriculture, Kinki University3327-204 Nakamachi, Nara, 631-8505, Japan; 2 BIOBANK Co. Ltd.388-1 Kita-ku, Okayama, 700-0952, Japan

**Keywords:** *Enterococcus faecalis* TH10, immunomodulatory effect, nitric oxide, RAW 264 cell, Toll-like receptor2

## Abstract

The objective of this study was to investigate the immunomodulatory effects of heat-killed *Enterococcus faecalis* TH10 (hk-TH10) and its signal transduction on murine macrophage RAW264 cells. RAW264 cells produced nitric oxide (NO) following hk-TH10 treatment. In order to investigate the mechanisms underlying hk-TH10-stimulated NO production, we further measured NO production in RAW264 cells treated with Toll-like receptor (TLR) 4 inhibitor peptide, NF-κB inhibitor, TLR1-siRNA, TLR2-siRNA, and TLR-6 siRNA. Furthermore, the activation of TLR2-TLR1/6 pathway molecules was analyzed by Western blotting. The result of this study showed that hk-TH10 stimulates NO in RAW264 cells through the activation of the TLR2-TLR1/6 pathway. From our findings, we can conclude that hk-TH10 isolated from a traditional side-dish fermented food (tempeh) may facilitate host immunomodulation.

## Introduction

Lactic acid bacteria in the gastrointestinal tract, such as lactobacilli and bifidobacteria, play an important role in the health of the host (Ohashi and Ushida 2009). In particular, these bacteria contribute to the modulation of immune responses (Won et al. 2011; Santos Rocha et al. 2012). Consumption of probiotic foods, mainly yogurt, has been reported to ameliorate abnormal immune functions, including allergy (Schiffrin et al. 2009; Chaves et al. 2011). Recent studies have found that not only viable cells of lactic acid bacteria but also heat-killed cells exhibit immunomodulatory effects (Liu et al. 2011; Kawase et al. 2012). Chang et al. (2007) and Ou et al. (2011) also reported that heat-killed lactic acid bacteria skewed the immune response toward T-helper 1 (Th1) polarization. Cell surface components in lactic acid bacteria, such as peptidoglycan, lipoproteins, and diacylated lipopeptide, act as ligands for immune cells (Takeuchi et al. 2002; Natsuka et al. 2008). Nitric oxide (NO) is involved in a variety of important physiological processes such as vasodilatation, neurotransmission, and host defense against invading pathogens (Bogdan 2001). However, an excessive amount of NO is detrimental, resulting in rheumatoid arthritis, gastritis, bowel inflammation, multiple sclerosis, and bronchitis (Abramson et al. 2001; Gassull 2004). In macrophages, NO is synthesized by the inducible isoform of nitric oxide synthase (iNOS), which catalyzes the conversion of l-arginine to l-citrulline and NO, in response to various stimuli such as lipopolysaccharide (LPS), interferon (IFN), tumor necrosis factor-α (TNF-α), and interleukin (IL)-1β (Nathan and Xie 1994). Peptidoglycan, lipoproteins, and diacylated lipopeptide also bind to Toll-like receptor (TLR) 2 and TLR1 or 6 heterodimers on the macrophage cell surface and then stimulate NO production via the canonical NF-κB pathway (Lorne et al. 2010; Wells 2011). Thus, cell surface components in lactic acid bacteria are thought to be immunomodulators.

In 1988, we isolated a lactic acid bacterium from tempeh, a traditional side-dish fermented food, which is widely consumed in Southeast Asia (Ohhira et al. 1988, 1990a). We further examined the biochemical properties of the isolated lactic acid bacterium and identified it as *Enterococcus faecalis* TH10 (Ohhira et al. 1988, 1990a). *Enterococcus faecalis is* generally present in the intestinal tract of humans and animals. This Gram-positive, nonmotile organism shows homolactic fermentation, among other characteristics (Ohhira et al. 1990b). Hitherto, we found an anti-*Escherichia coli* O-157 component produced by *E. faecalis* TH10 (Ohhira et al. 2000). In this study, we showed the immunomodulatory effect of heat-killed *E. faecalis* TH10 (hk-TH10) and its signal transduction on murine macrophage RAW 264 cells.

## Materials and Methods

### Preparation of heat-killed *Enterococcus faecalis* TH10

*Enterococcus faecalis* TH10 was cultured in glucose–yeast–peptone medium at 37°C for 24 h. After incubation, the TH10 cells were harvested by centrifugation at 3000 × *g* for 20 min. The cells were washed twice with sterilized phosphate-buffered saline (0.85% NaCl, 2.86 mmol/L KCl, 10 mmol/L Na_2_HPO_4_, and 1.76 mmol/L KH_2_PO_4_ at pH 7.7) and then heat-killed at 121°C for 15 min by autoclaving.

### Reagents

Antibodies against p44/42 mitogen-activated protein kinases (MAPKs) or extracellular signal-regulated kinase (ERK), phospho-p44/42 MAPK (Thr202/Tyr204; p-ERK), p38 MAPK, phospho-p38 MAPK (Thr180/Tyr182; p-p38), stress-activated protein kinase (SAPK)/c-Jun NH_2_-terminal protein kinase JNK (JNK), phospho-SAPK/JNK (Thr183/Tyr185; p-JNK), iNOS, COX2, Stat1, phospho-Stat1, IκBα, and NF-κB (p65) were obtained from Cell Signaling Technology (Beverly, MA, USA). Antibody against β-actin was purchased from Sigma-Aldrich (St. Louis, MO, USA). The TLR4 peptide inhibitor set VIPER was purchased from IMGENEX (San Diego, CA, USA). The NF-κB inhibitor ammonium pyrrolidine dithiocarbamate (APDC) was purchased from Merck Millipore (Darmstadt, Germany).

### Cell culture and treatment

Murine macrophage RAW264 cells were purchased from RIKEN Bio Resource Center (Tsukuba, Japan). Cells were cultured in Dulbecco's Modified Eagle's Medium containing 10% heat-inactivated fetal bovine serum (Invitrogen, Carlsbad, CA, USA), 100 U/mL of penicillin, and 100 μg/mL streptomycin in a humidified atmosphere of 5% CO_2_ at 37°C. RAW 264 cells (2 × 10^5^ cells/well) were seeded into a 24-well multiplate and cultured for 12 h. Hk-TH10 (5–100 μg/mL) or LPS (final concentration; 200 ng/mL; Sigma-Aldrich) was then added to the cells, and culturing was continued for 24 h.

### Small-interfering RNA experiments

Before transfection, RAW264 cells (2 × 10^5^ cells/well) were seeded into a 24-well multiplate and cultured for 12 h. Four small-interfering RNA duplexes (siRNAs) specific for mouse *TLR1*, *TLR2*, and *TLR6* genes and a nonsilencing control siRNA without any homology to known mouse genes were synthesized by Sigma-Aldrich. RAW264 cells were transfected with each of the siRNAs by using the FuGENE® HD Transcription reagent (Promega, Madison, WI, USA). After an additional incubation of 48 h at 37°C, the downregulation levels of *TLR1, TLR2*, and *TLR6* were assessed by Western blot analyses using anti-TLR1, -TLR2, and -TLR6 antibodies. After siRNA transfection, the RAW 264 cells were stimulated with hk-TH10 (25 μg/mL) and cultured for 24 h.

### Measurement of NO production

After stimulation with LPS or hk-TH10 for 24 h, cell culture media were collected and then centrifuged at 4°C for 5 min. A Griess reagent kit (Promega) was used to measure the amount of nitrite, a stable metabolite of NO, in the supernatants. Briefly, 50 μL of each culture medium was added to 96-well multiplate in triplicate, and the same volume of sulfanilamide solution was dispensed. After incubation at room temperature for 10 min, dispensed 50 μL of *N*-1-naphthylethylenediamine dihydrochloride solution was dispensed to all wells. After incubation at room temperature for 10 min, the absorbance was measured at 540 nm using a colorimetric microplate reader (Corona Grating Microplate Reader SH-9000; Corona Electric Co., Ltd., Hitachinaka, Japan).

### Quantitative real-time PCR

Total RNA was extracted from cells using TRIzol reagent (Invitrogen) followed by DNase I treatment. cDNA was synthesized from 0.25 μg of total RNA using a PrimeScript reagent kit (TaKaRa Bio, Ohtsu, Japan). cDNA was subjected to quantitative real-time PCR using a StepOne Real-Time PCR system (Applied Biosystems). Primers for *iNOS, COX-2,* and the gene-encoding *glyceraldehyde-3-phosphate dehydrogenase* (*GAPDH*) were purchased from TaKaRa Bio. The expression level of each gene was determined using the comparative *C*_t_ method and normalized to that of *GAPDH*, an internal control. The PCR reaction consisted of 45 cycles (95°C for 10 s and 60°C for 40 s) after an initial denaturation step (95°C for 10 min).

### Western blotting

Whole-cell extracts were prepared by lysis of cells in RIPA buffer (10 mmol/L Tris–HCl (pH 7.5), 150 mmol/L NaCl, 1 mmol/L ethylenediamine-*N*,*N*,*N*′,*N*′-tetraacetic acid, 1% NP-40, 0.1% sodium deoxycholate, and 0.1% sodium dodecyl sulfate [SDS]) containing complete protease and phosphatase inhibitor cocktails (Roche, Penzberg, Germany). Samples were subjected to SDS-polyacrylamide gel electrophoresis and electroblotted onto PVDF membranes. The membranes were incubated with a primary antibody, followed by incubation with a horseradish peroxidase-conjugated secondary antibody. Immunolabeled proteins were detected using an ECL chemiluminescence kit (GE Healthcare, Piscataway, NJ, USA) and a LAS-4000 lumino-image analyzer (Fuji Film, Tokyo, Japan).

### Statistical analysis

All data were analyzed using Student's *t*-test or one-way ANOVA followed by Fisher's multiple range test.

## Results

### Heat-killed *Enterococcus faecalis* TH10 stimulates NO production in RAW 264 cells

**Figure 1:**
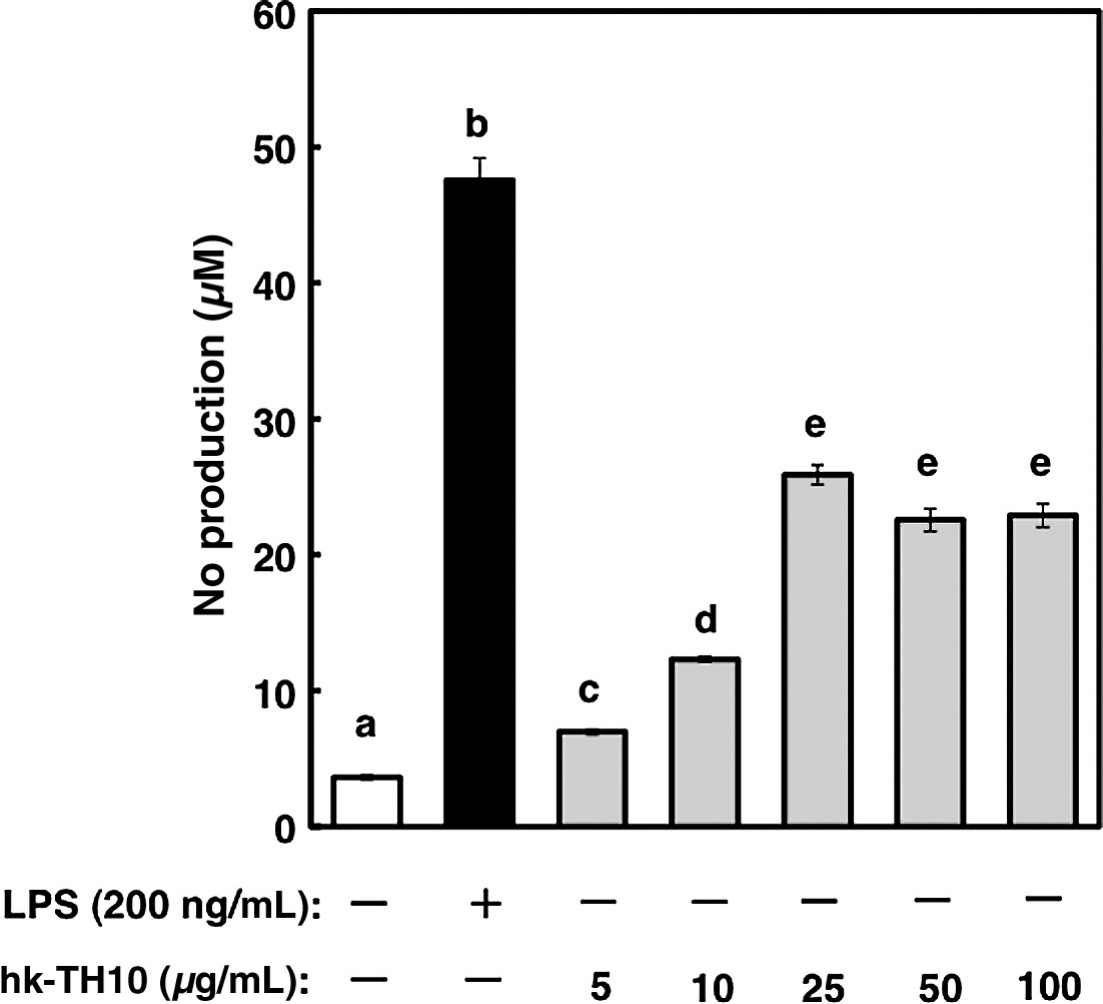
Effects of hk-TH10 treatment on nitric oxide (NO) release from RAW264 macrophage cells. RAW264 macrophage cells were incubated for 24 h in the presence or absence of hk-TH10. After incubation for 24 h, cell culture media were harvested for measurement of nitrite, a stable metabolite of NO (mean ± SE, *n* = 12). Statistical significance was determined by one-way ANOVA and Fisher's multiple range test (*P* < 0.05).

The concentration of nitrite was measured as an index of NO production. As shown in Figure 1, NO was markedly stimulated by hk-TH10. The NO production level gradually increased as the concentration of hk-TH10 increased to 25 μg/mL, but the NO production levels did not change at concentrations above 25 μg/mL. The NO production level at 25 μg/mL hk-TH10 was approximately 50% of NO production level at 200 ng/mL LPS.

**Figure 2:**
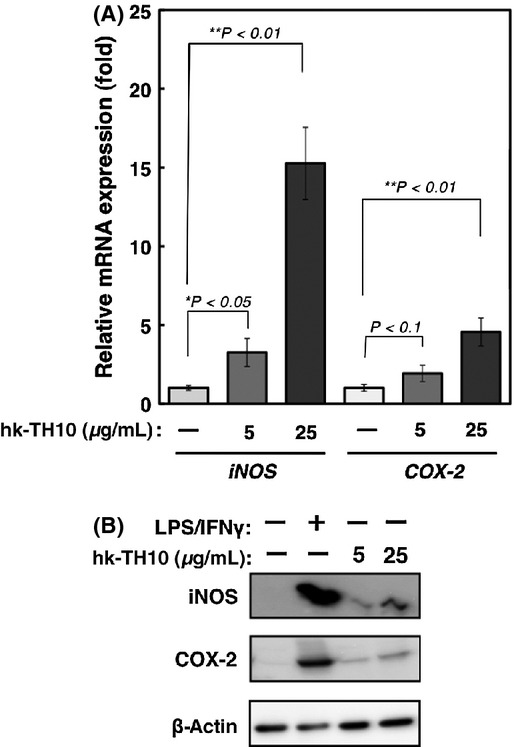
Effects of hk-TH10 treatment on the expression of pro-inflammatory genes in RAW264 macrophage cells. RAW264 macrophage cells were incubated for 24 h in the presence or absence of hk-TH10. (A) After incubation in the presence or absence of hk-TH10 for 12 h, total RNA was harvested and subjected to quantitative RT-PCR for *iNOS* and *COX-2* (mean ± SE, *n* = 9). Asterisks indicate statistical significance as determined by Student's *t*-test (*P < 0.05, **P < 0.01). (B) After incubation, cell lysates were harvested and subjected to Western blot analysis for iNOS, COX-2, and β-actin. A representative blot from three independent experiments is shown.

To gain more information regarding the mechanisms by which hk-TH10 stimulates NO production, we examined the mRNA and protein expression levels of iNOS and COX-2 by using quantitative real-time PCR and Western blot analyses, respectively. Both *iNOS* and *COX-2* mRNA levels were upregulated by hk-TH10 treatment (Fig. 2A), thereby upregulating both iNOS and COX-2 protein levels (Fig. 2B). The mRNA and protein expression levels of iNOS and COX-2 were upregulated in a dose-dependent manner.

### Heat-killed *Enterococcus faecalis* TH10-stimulated NO production is not mediated through the TLR4 pathway

**Figure 3:**
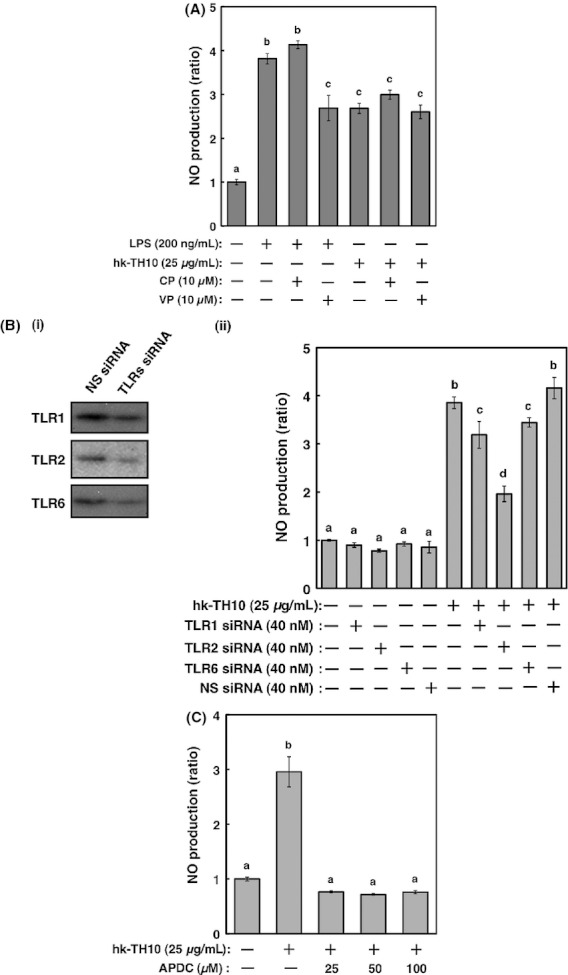
Nitric oxide (NO) production stimulated by hk-TH10 is mediated through the Toll-like receptor (TLR)2-TLR1/6 pathway. (A) RAW264 macrophage cells were pretreated with TLR4 inhibitor (VP; 10 μmol/L) or inert control peptide for VP (CP; 10 μmol/L) for 3 h, and then cultured with lipopolysaccharide (200 ng/mL) or hk-TH10 (25 μg/mL). (B) (i) Western blot analysis. The expression levels of TLR1, TLR2, and TLR6 proteins at 48 h after transfection were determined. A representative blot from three independent experiments is shown. (ii) RAW264 macrophage cells were transfected with nonsilencing (NS)-siRNA, TLR1-siRNA, TLR2-siRNA, or TLR6-siRNA (40 nmol/L each). After transfection for 24 h, hk-TH10 was added to the culture media, and culturing was continued for 24 h. (C) RAW264 macrophage cells were pretreated with NF-κB inhibitor (ammonium pyrrolidine dithiocarbamate; 25–100 μmol/L) for 2 h, and then cultured with hk-TH10 (25 μg/mL) for an additional 24 h. The cell culture media were harvested for measurement of nitrite, a stable metabolite of NO (mean ± SE, *n* = 24). Statistical significance was determined by one-way ANOVA and Fisher's multiple range test (*P* < 0.05).

To understand the mechanisms underlying hk-TH10-stimulated NO production, we first examined whether hk-TH10-stimulated NO production was mediated through the TLR4 pathway by using a TLR4 inhibitor. LPS-stimulated NO production was significantly suppressed by TLR4 inhibitor (VIPER (VP, amino acid sequence of the viral inhibitory peptide of TLR4 : KYSFKLILAEYRRRRRRRRR, molecular weight: 2780.3)) treatment (Fig. 3A). It is well known that LPS binds to the cell surface receptor CD14, which triggers activation of TLR4 and downstream signaling molecules such as IκBα and MAPKs including JNK, p38 MAPK, and ERK, culminating in the expression of pro-inflammatory genes such as *iNOS*, *COX-2*, *TNF-α,* and *IFN-β* (Lorne et al. 2010; Wells 2011). In contrast, the hk-TH10-stimulated NO production was not changed by TLR4 inhibitor. The CP (inert control peptide for VP amino acid sequence of the control peptide: RNTISGNIYSARRRRRRRRR, molecular weight: 2601) treatment did not suppress the LPS-stimulated or hk-TH10-stimulated NO production. From these results, we can conclude that hk-TH10-stimulated NO production is not mediated through the TLR4 pathway.

### Heat-killed *Enterococcus faecalis* TH10-stimulated NO production is mediated through the TLR2-TLR1/6 pathway

It is well known that peptidoglycan, lipoproteins, and diacylated lipopeptide, which are surface components of lactic acid bacteria, bind to TLR2 and TLR1/6 on the macrophage cell surface and then produce NO via the canonical NF-κB pathway. In order to investigate whether hk-TH10-stimulated NO production is mediated through the TLR2-TLR1/6 pathway, we examined whether knockdown of endogenous TLR1, TLR2, and TLR6 by each siRNA affected hk-TH10-stimulated NO production. These TLR1-, TLR2-, and TLR6-siRNA treatments significantly suppressed hk-TH10-stimulated NO production. In particular, the level of NO production in TLR2-siRNA-treated RAW264 cells was lower than that in TLR1- or TLR6-siRNA-treated RAW264 cells (Fig. 3B). Conversely, there was no effect on hk-TH10-stimulated NO production in the nonsilencing control siRNA-treated RAW264 cells. Next, we further examined whether the NF-κB inhibitor APDC (25–100 μmol/L) could affect hk-TH10-stimulated NO production. The NF-κB inhibitor treatment greatly suppressed NO production (Fig. 3C).

**Figure 4:**
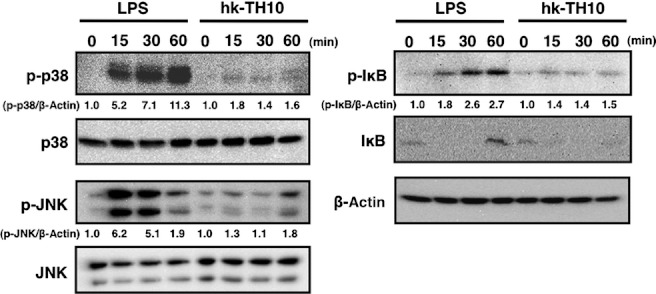
Effects of hk-TH10 on signal transduction in RAW 264 macrophage cells. RAW 264 macrophage cells were stimulated by lipopolysaccharide or hk-TH10. After incubation for the indicated periods, the cell lysates were harvested and analyzed by Western blot analysis for the indicated proteins. A representative blot from three independent experiments is shown.

TLR2-TLR1/6 signaling enhances the phosphorylation of MAPKs and IκBα, and thereby activates transcription factors such as AP-1, ELK1, and NF-κB. These transcription factors are activated or upregulated by peptidoglycan, lipoproteins, and diacylated lipopeptide, which bind to the iNOS promoter and enhance NO production. Hk-TH10 stimulation enhanced phosphorylation of MAPKs including p38 (Fig. 4, left panel). Phosphorylation of IκBα proteins leads to its degradation and the translocation of NFκB into the nucleus. As shown in Figure 4 (right panel), hk-TH10 stimulation facilitated the phosphorylation of IκBα. Taken together, these results suggest that hk-TH10-stimulated NO production is mediated through the TLR2-TLR1/6 pathway. The activation levels of each molecule were lower than those in LPS-stimulated RAW264 cells.

## Discussion

**Figure 5:**
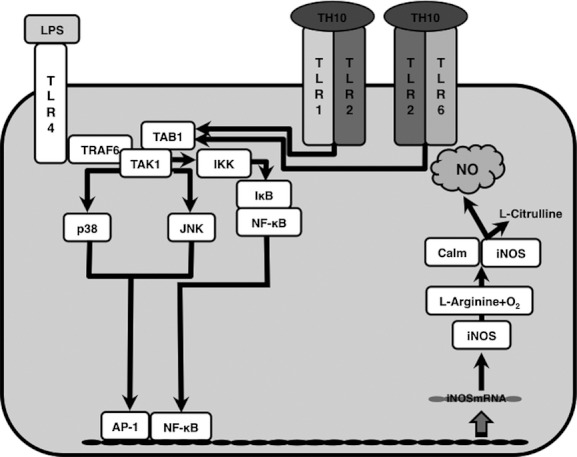
A schematic diagram showing the nitric oxide production pathway induced by hk-TH10 treatment in RAW264 cells. Activation of the Toll-like receptor (TLR)2-TLR1/6 pathway by hk-TH10 induces the expression of iNOS and COX-2.

In this study, we showed that the immunomodulatory effects of hk-TH10 are mediated via the TLR2-TLR1/6 pathway (Fig. 5). Ten TLRs have been identified in humans and 12 in mice (Takeda et al. 2003; Kawai and Akira 2010; Yamamoto and Takeda 2010). TLR1, 2, 4, 5, 6, and 11 recognize microbial membrane components, whereas TLR3, 7, 8, and 9 recognize nucleic acid of bacteria and virus (Takeda et al. 2003; Kawai and Akira 2010; Yamamoto and Takeda 2010). Cell wall components of lactobacilli can bind to TLR2 in combination with TLR6 (Natsuka et al. 2008; Wells 2011). The diacyl lipid chains of diacylated lipoprotein bind to a hydrophobic pocket in the extracellular domain of TLR2 (Jin et al. 2007; Jin and Lee 2008). In addition, the third lipid chain of triacylated lipoprotein binds to the hydrophobic pocket in TLR1 (Jin et al. 2007; Jin and Lee 2008). Because of these interactions, MyD88/TRAF6/TAK1 signaling is activated, leading to the activation of MAPKs and the canonical NF-κB pathway. In this study, NO production levels in RAW264 cells were significantly suppressed by TLR1-, TLR2-, or TLR6-siRNA treatments and by NF-κB inhibitor APDC treatment. These results suggest that NO production stimulated by hk-TH10 is mediated through the TLR2-TLR1/6 pathway. Moreover, the degree of immunomodulation in hk-TH10-stimulated RAW264 cells was distinctly lower than the degree of that caused by LPS stimulation. Recent studies have shown that the production levels of pro-inflammatory cytokines such as TNF-α, IL-1β, and IL-6 differ among lactic acid bacteria strains or between viable and heat-killed cells of lactic acid bacterium (Suzuki et al. 2008; Chon et al. 2009). TLR and cytokine expression levels after stimulation with heat-killed Gram-positive and Gram-negative bacteria are different, as is their dose-dependent mechanism of action (Beran et al. 2011). From these reports, we thought that the amounts and thermostability of active components in the cells could contribute to differences in macrophage activation. We also thought that this modest activation in hk-TH10-stimulated RAW264 cells might be good for the health of the host because an excessive amount of NO induces inflammatory diseases.

In conclusion, hk-TH10 stimulates NO in RAW 264 cells through the activation of the TLR2-TLR1/6 pathway. NO plays an important role in NK cells, mast cells, and neutrophil cells in the production of several cytokines (Tripathi 2007; Tripathi et al. 2007). These cytokines initiate immune responses such as bacterial cell killing and T or B cell proliferation (Calder 2007; Diveu et al. 2008). Thus, hk-TH10 may be useful in foods for the facilitation of host immunomodulation.
